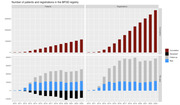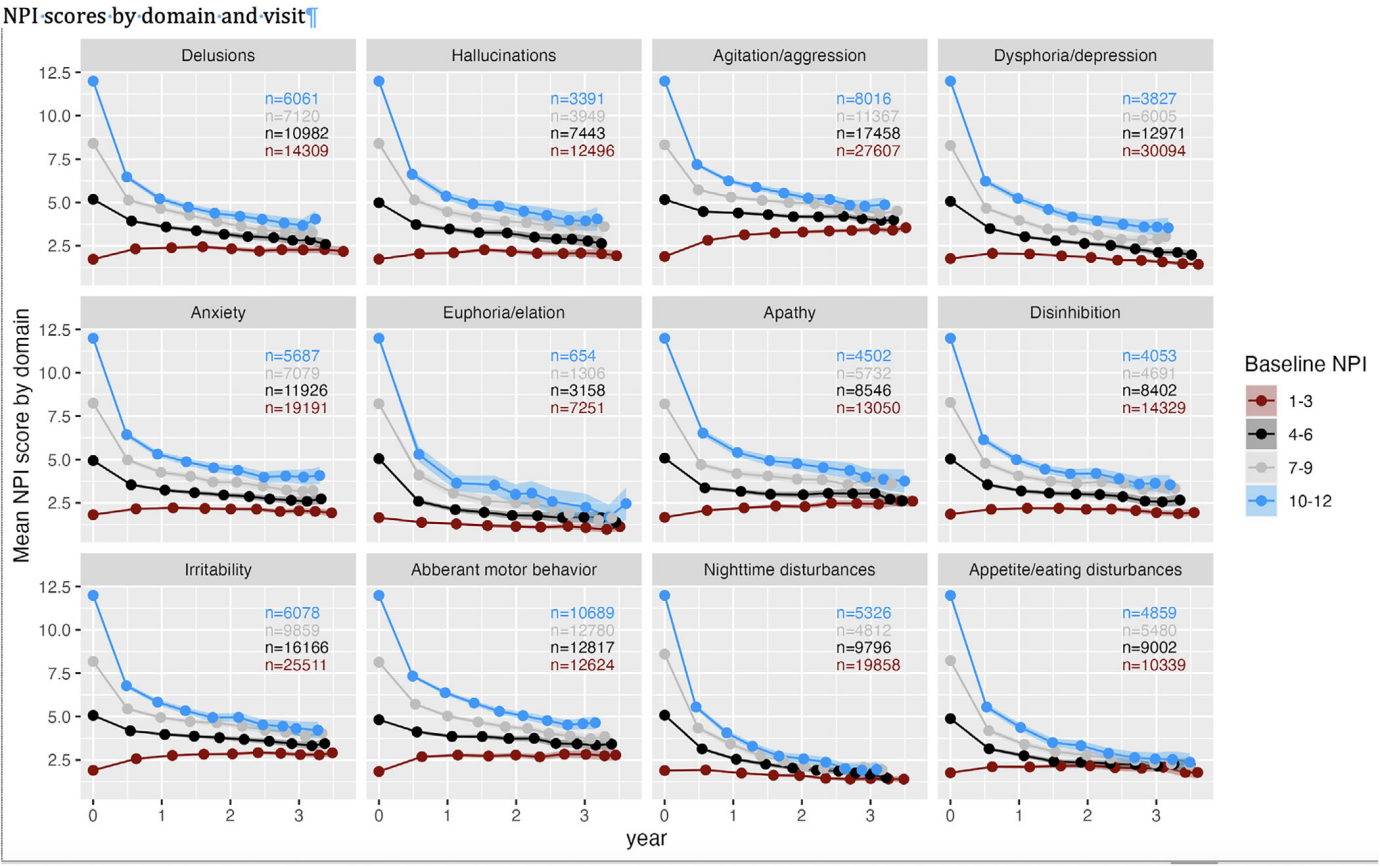# The Swedish registry for behavioural and psychological symptoms of dementia (BPSD)

**DOI:** 10.1002/alz.087866

**Published:** 2025-01-09

**Authors:** Linus Jönsson, Elisabet Londos, Moa Wibom, Katarina Nägga

**Affiliations:** ^1^ Karolinska Institutet, Solna, Stockholm Sweden; ^2^ Lund University, Malmö, Skåne Sweden; ^3^ Ängelholm Hospital, Ängelholm Sweden; ^4^ Linköping University, Linköping Sweden

## Abstract

**Background:**

Behavioural and psychological symptoms (BPSD) are common in major cognitive disorders and an important driver of suffering and high care needs. The Swedish BPSD registry was founded in 2010 to develop an evidence base for quality improvement in the care of patients with BPSD. Here we describe the process of establishing and operating the registry, the patient population included, and data collected since the start of the registry in 2010.

**Methods:**

The Swedish BPSD registry provides a framework for documenting the occurrence of behavioural disturbances, formulating individual caring and treatment plans and following up the patients and capturing health outcomes. Symptoms are recorded by the neuropsychiatric inventory ‐ nursing home (NPI‐NH) scale, and data is entered by trained staff, mainly at institutional care facilities. Triggering factors for BPSD are also recorded, as well as care objectives and targeted symptoms.

**Results:**

Between 2010 and 2023, 324,900 registrations were made in 110,684 persons. The number of patients active in the registry each year is 20‐25,000. Alzheimer’s disease was the most common diagnosis, 33%, followed by vascular dementia 17%, mixed 8%, DLB/PDD in 3% and FTD in 1,5%. Pain was the most cited trigger factor in all symptoms, except for night‐time and appetite/eating disturbances. The most frequently used care objective was affirmation/security, especially to manage agitation/aggression. Median survival was 24.0 months for men, 30.5 months for women.

**Conclusions:**

The Swedish BPSD registry provides a unique view of the final stages in the disease course of dementia, with specific focus on the role of behavioural disturbances and their management. The scale, data completeness, long duration and possibility of linkage to other health registries is to our knowledge unparalleled and a great potential as a resource for data‐driven research.